# Effect of community level intervention on nutritional status and feeding practices of under five children in Ile Ife, Nigeria

**DOI:** 10.11604/pamj.2015.22.255.7121

**Published:** 2015-11-18

**Authors:** Olorunfemi Akinbode Ogundele, Tolulope Ogundele

**Affiliations:** 1 Department of Community Health, Obafemi Awolowo University Teaching Hospital Complex, Ile –Ife, Osun State, Nigeria; 2 Department of Paediatrics, Obafemi Awolowo University Teaching Hospital Complex, Ile –Ife, Osun State, Nigeria

**Keywords:** Nutritional status, CIMCI, weight for age, MUAC, complementary feeding

## Abstract

**Introduction:**

Childhood malnutrition remains a widespread problem in developing world like Nigeria. The country ranks second among the ten countries contributing to sixty percent of the world's wasted under-five children. Community Integrated Management of Childhood illness (CIMCI) is a programme that employs the use of community based counsellors to address child health and nutritional challenges of the under-five and has the potential to reduce the morbidity and mortality resulting from poor nutritional and feeding practices. The study assessed the effect of community level intervention on nutritional status and feeding practices of children in Ile-Ife, Nigeria.

**Methods:**

A cross-sectional comparative study that employed the use of multi stage cluster sampling techniques in selecting 722 mothers of index under five children. The study was done in two Local Government Areas of Osun State, Nigeria. Quantitative techniques were used in data collection. Data analysis was done using SPSS version 20.0. Descriptive and bivariate analyses was performed.

**Results:**

The two Local Government Area (LGA) did not differ significantly in their wealth index (p = 0.344). However, more children in the non-implementing LGA (16.1%) had low weight for age compared with 3.6% in the CIMCI implementing LGA (p = 0.000). A statistically significant difference exist in the MUAC measurement of children 12-23 months between the CIMCI implementing and non-implementing communities (p = 0.007). A higher percentage of caregivers (19.3%) introduced complementary feeding earlier than 6 months in the non-implementing area (p < 0.001).

**Conclusion:**

Using community level nutritional counseling can greatly improve nutritional status and feeding practices of under five children.

## Introduction

Child undernutrition is a major public health problem in Nigeria like in other Sub-Saharan African countries. Malnutrition is a direct or indirect cause of 54% of all childhood death [1]. In addition to this mortality risk, there is early growth retardation with delayed motor development [2] and impaired cognitive function and school performance [3]. In Africa, almost two out of five children are stunted, an estimated total of 60million children. Nigeria alone accounts for 11 million of these children [4, 5]. Report from the Nigerian Demographic Health Survey (NDHS) 2008 indicate that Forty one percent of children under five years are chronically malnourished (i.e. stunted), and 23% of children suffer from severe stunting. Fourteen percent of children under-five in Nigeria are wasted and 7% are severely wasted, an increase from 11% wasting and 4% severe wasting obtained in 2003 NDHS [6]. The importance of care giving behaviours was recognized by UNICEF in its conceptual model of factors that determine children's risks of malnutrition, death and disability [7]. More specifically, successful nutritional and feeding practices requires not only that foods of adequate energy and nutrient quality be available, but also a range of appropriate behaviours by the caregiver. Community Integrated Management of Childhood illness (CIMCI) is a World Health Organisation programme that employs the use of community based counsellors to address some of these challenges and has the potential to reduce mortality. The nutritional impact of this initiative by World Health Organisation has been established. A study in Honduras revealed that the prevalence of severe malnutrition in an intervention population decreased from 28% to 4% within 2 years of implementation. The improvements were sustainable for another three years after the programmes left the intervention area. Children in intervention communities continued to do better than those in comparison communities and that the younger siblings were better nourished than age matched controls [8]. CIMCI was implemented in Ife Central Local Government Area in Osun state, Nigeria in 2005. Community Resource Persons (CORPs) were trained to provide information to caregivers on appropriate nutritional and child care practices. They also ensured that the practices were adopted as much as possible. These CORPs have now operated for more than six years in these communities to promote appropriate nutritional practices for optimal growth and development of under five children. This study was conducted to assess the effect of community level intervention on nutritional status and feeding practices of children in Ile-Ife, Nigeria.

## Methods

**Study location**: The study was done in one CIMCI implementing and one non-CIMCI implementing Local Government Area (LGA) in Osun State, Nigeria. The state has a population of about 3.4 million [9] people. Ife Central local Government Area the study site was purposively chosen because its implements CIMCI. It has trained CORPs who work as community based counsellors. The comparison site is Ilesa East Local Government Area. The LGA does not implement the CIMCI programme and has no trained CORPs.

**Study population**: The study populations were mothers of children 0-59 months of age, and their index children.

**Study design**: the study was a comparative cross-sectional design.

**Sample size estimation**: Sample size was determined using the formula for comparing independent proportions [10]. This gave a sample size of 722 mothers of index under five children. Three hundred and sixty-one respondents were selected from each LGA.

**Sampling Technique**: A multi stage cluster sampling technique was employed in selection of study subjects as follows. In stage 1, Ife Central LGA, the study site was purposively selected among the thirty LGAs in the state being the only LGA that implements CIMCI. Ilesa East LGA, the comparison LGA was randomly selected among the remaining twenty-nine non CIMCI implementing LGAs in the State. In stage 2, Two implementing ward 4 and 5 were purposively chosen in Ife Central LGA. Wards 2 and 8 were randomly selected in Ilesa East among the 11 wards in the LGA. In stage 3, Enumeration Areas (EAs) in each selected ward were used as primary sampling unit. The selected ward in Ife Central had 180 EAs while those in Ilesa East had 150 EAs. One-fifth (1/5) of the EAs were selected using simple random selection method thus 36 EAs were selected from wards 4 and 5 in Ife Central and 30 EAs were selected from wards 2 and 8 in Ilesa East. In each LGA the sample size of 361 was divided by the selected number of enumerated area to determine the average number of respondents to recruit from each EA. An average of 10 respondents were recruited from selected EAs in wards 4 and 5 in Ife Central and 12 respondents from the selected EAs in wards 2 and 8 in Ilesa East until the sample size of 361 was obtained. In stage 4, household listing of the streets in the selected enumeration area was done. One street was randomly selected from each EA. In the selected street an eligible household is randomly selected as the starting point. Subsequent household selection is done by systematic random sampling. Interview began in the selected eligible household until the targeted number of interviews has been obtained. In stage 5, one mother with an eligible child was interviewed in every household selected. In any visited household the eligibility for participation was the presence of a child 0-59 months. In situations where the household had more than one eligible subject the child with birthday nearest to the interview period was chosen. Where there was no eligible child in the household the next household with an eligible child was chosen. Excluded were households without children, or mothers who did not give consent or whose children were ill during the study.

**Data collection**: A structured interviewer administered pre-tested questionnaire was used in data collection. Data was collected on the family, social, demographic and household characteristics, infant feeding practices, immunization history and recent episodes of acute illnesses in the children. The questionnaire was adapted from the CIMCI household level survey questionnaire developed by United Nation Children's Funds (UNICEF) and previously validated by WHO/Nigeria [11]. The questionnaire were administered in English or Yoruba depending on the respondents′ preference.

### Measurements

**Weight**: Standardized and calibrated stand-on scales, the electronic bathroom scale type were used to weigh children 0-59 months in their underpants. For children who could not stand, the mother was weighed together with the child, then the mother was weighed alone and the difference was recorded as the child's weight and measured in kilograms. The scale was checked before each weighing to ensure that the mark returned to zero. Each child was weighed twice.

**Mid Upper Arm Circumference (MUAC)**: MUAC of children 12-59 months was done on the left arm using Shakir's strip. The Shakir's tape measure is a plastic tape measure that has been standardized and adapted by UNICEF and used in community nutritional surveys. The mid upper arm circumference was taken at the level of the upper arm midpoint between the tip of the scapula (acromion process) and the olecranon process.

**Statistical Analysis**: The data was analyzed using SPSS version 20. Anthropometric measurements were converted, using CDC/WHO Epi-Info 6.0 software to standard z-score, and exported back to SPSS. Underweight was defined as z-scores of -2 or less for the Child Growth Standards reference value for age (World Health Organisation) [12]. Mid-upper arm circumference cut off was set at 12.5cm for normal nutritional status. MUAC range of < 12.5cm - 11.5cm was taken as moderate malnutrition while MUAC < 11.5cm was taken as severe malnutrition. This corresponds with the green, yellow and red colour of Shakir's strip used in the measurement of MUAC. Wealth Index was constructed by assessing the presence or absent of durable assets in the household. The questions used to establish the wealth index included household access to electricity, radio or television; household ownership of bicycle, motorcycle or car; type of material used for flooring the house; number of rooms in the house; main source of drinking water; type of toilet facility. The presence was scored as 1 and the absence as 0, the mean assets score was re-categorized into five different wealth quintiles of equal proportion (Lowest, second, third, fourth and Highest wealth quintiles) using principal component analysis. Appropriate univariate and bivariate analyses were done. Chi-square test or Fishers exact test was used to compare for possible significant differences between respondents in CIMCI implementing and non-implementing LGAs. Where the numbers of cells were more than four, the Likelihood chi-square test was used. A p-value of less than 0.05 was regarded as statistically significant

**Ethical clearance**: Ethical approval was obtained from the ethics and research committee of Obafemi Awolowo University Teaching Hospitals Complex. Confidentiality was maintained, and the right not to participate or withdraw at any time in the study was assured.

## Results

Seven hundred and twenty two mother-child pair was recruited for the study. The socio-demographic characteristics of the children′s parents are as shown in Table 1. Most of the respondents in both LGAs were married, over 90% in both LGA. A higher proportion of mothers (39%) in the non-CIMCI implementing LGA had tertiary education compared with 24.1% in the CIMCI implementing LGA. About 69% of respondents had family size less than five in the CIMCI implementing area compared 81.7% in the non-implementing area. No statistically significant difference exists in the wealth index between the two LGA (p = 0.344).

**Table 1:** Socio-demographic characteristics of respondents and index child

Characteristics	CIMCI implementing LGA	NON-CIMCI LGA	Statistical Indices
	N = 361 n (%)	N = 361 n (%)	
**Maternal Age yrs**			
15-19	4 (1.1)	2 (0.6)	*χ^2^ = 1.56,df = 3 P = 0.669
20-29	174 (48.2)	168 (46.5)	
30-39	158 (43.8)	170 (47.1)	
40-49	25 (6.9)	21 (5.8)	
**Marital Status**			
Married	339 (93.9)	348 (96.4)	χ^2^ = 2.41 df = 2 P = 0.119
Not married	22 (6.1)	13 (3.6)	
**Maternal Education Status**			
No Education	15 (4.2)	10 (2.8)	*χ^2^ = 32.77 df = 3 P< 0.001
Primary Education	72 (19.9)	28 (7.8)	
Secondary Education	187 (51.8)	183 (50.7)	
Tertiary Education	87(24.1)	140 (38.8)	
**Maternal Occupation**			
Trader/Self employed	313 (86.7)	273 (75.6)	*χ^2^ = 36.31 df = 3 P< 0.001
Farmers	13 (3.6)	3 (0.8)	
Housewife	18 (5.0)	39 (10.8)	
Government Employee	17 (4.7)	46 (12.7)	
**Index Children Age (months)**			
<5	29 (8.0)	55 (15.2)	*χ^2^ = 34.59 df = 4 P= 0.001
6-11	47 (13.0)	82 (22.7)	
12-23	61 (16.9)	75 (20.8)	
24-35	114 (31.6)	70 (19.4)	
36-59	110 (30.5)	79 (21.9)	
**Sex**			
Male	209 (57.9)	195 (54.0)	χ^2^ = 1.10 df = 1 P= 0.290
Female	152 (42.1)	166 (46.0)	
**Family size**			
< 5	248 (68.7)	295 (81.7)	χ^2^ = 16.41, df = 1 P< 0.001
> 6	133 (31.3)	66 (18.3)	
**Wealth Index**			
First and Second	234(64.8)	246(68.1)	χ^2^ = 0.89 df = 1 P= 0.344
Third and above	127(35.2)	115(31.9)	

* Likelihood Chi square test, CIMCI = Community Integrated Management of Childhood Illness, Local Government Area

A higher proportion of index children in the non-implementing LGA (16.1%) had low weight for age compared with 3.6% in the CIMCI implementing LGA (χ^2^=31.6, p = 0.000). For all age groups there were more children with “low weight for age“ in the non-implementing LGA compared to the CIMCI-implementing LGA. For children in age group less than 12 months, 30.7% of those in the non-implementing LGA had low weight for age compared to 17.1% in the CIMCI-implementing LGA, this difference was statistically significant (χ^2^=4.68, p = 0.030). For the rest of the age groups in the CIMC-implementing LGA, the children had normal weight for age compared to 13.3%, 2.9%, 7.0% and 2.8% with low weight for age observed in corresponding age groups in the non-implementing LGA. The difference was not statistically significant except for age groups, 12-23 months (χ^2^= 6.93, p = 0.008). More males than females had low weight for age in both implementing and non-implementing LGAs (Table 2).

**Table 2:** Nutritional status of index children by age and sex

Variable	CIMCI implementing LGA N= 361			NON-CIMCI LGA N = 361			Statistical indices	
	LWFA	NWFA	Total	LWFA	NWFA	Total	χ^2^	P
Age in months	n (%)	n (%)	n (%)	n (%)	n (%)	n (%)		
<12	13 (17.1)	63 (82.9)	76 (100.0)	42 (30.7)	95 (69.3)	137(100.0)	4.68	**0.030**
12-23	0 (0.0)	61 (100.0)	61 (100.0)	10 (13.3)	65 (86.7)	75 (100.0)	*6.93	**0.008**
24-35	0 (0.0)	114(100.0)	114(100.0)	2 (2.9)	68 (97.1)	70 (100.0)	*1.18	0.276
36-47	0 (0.0)	55 (100.0)	55 (100.0)	3 (7.0)	40 (93.0)	43 (100.0)	*1.97	0.162
48-59	0 (0.0)	55 (100.0)	55 (100.0)	1 (2.8)	35 (97.2)	36 (100.0)	*0.05	0.830
								
**Index child had weight low for age**								
Yes	13(3.6)			58(16.1)			31.6	**0.000**
No	348(96.4)			303(83.9)				
**Sex**								
Male	10 (4.8)	199 (95.2)	209(100.0)	39 (20.0)	156 (80.0)	195(100.0)		
Female	3 (2.0)	149 (98.0)	152(100.0)	19 (11.4)	147 (88.6)	166(100.0)		

LWFA = Low weight for age, NWFA = Normal weight for age

* Fisher's exact test significant at P < 0.05 CIMCI = Community Integrated Management of Childhood Illness, LGA= Local Government Area.

As regards MUAC of children 12-59 months, MUAC< 12.5cm was observed only in the age group 12-23 months in the CIMCI-implementing LGA, (6.5%). Of this 6.5% in the CIMCI-implementing LGA, 1.6% was severely malnourished (MUAC < 11.5cm) and 4.9% moderately malnourished (< 12.5cm-11.5cm). In the non-implementing LGA on the other hand 26.7% had MUAC < 12.5cm. Of these 26.7% in the non-implementing LGA 6.5% was severely malnourished (MUAC < 11.5cm) and 20% was moderately malnourished (MUAC < 12.5cm-11.5cm). This difference was statistically significant (p = 0.007). Among those in the age groups 24-35 and 36-47 months in the non-implementing LGA, 7.1% and 2.3% were severely malnourished (MUAC <11.5cm) respectively, while none was malnourished in children of same age group in the CIMCI-implementing LGA. The different was also statistically significant in the age group 24-35 between the two LGA (p = 0.000). None of the children in the age group 48-59 months was malnourished in both implementing and non-implementing LGAs. However in both LGAs more males than females were malnourished (Table 3).

**Table 3:** Nutritional status of index children aged 12 - 59 months based on MUAC

CIMCI implementing LGA N = 285					NON-CIMCI LGA N = 224			
								
**MUAC**	<11.5cm	11.5 < 12.5cm	>12.5cm		<11.5cm	11.5 < 12.5cm	>12.5cm	
	Severe	Moderate	Normal	Total	Severe	Moderate	Normal	Total
**Age in months**	n (%)	n (%)	n (%)	n (%)	n (%)	n (%)	n (%)	n (%)
12-23	1(1.6)	3 (4.9)	57 (93.4)	61 (100.0)	5 (6.7)	15 (20.0)	55 (73.3)	75 (100.0)
24-35	0 (0.0)	0 (0.0)	114(100.0)	114(100.0)	5 (7.1)	11(15.7)	54 (77.2)	70 (100.0)
36-47	0 (0.0)	0 (0.0)	55 (100.0)	55 (100.0)	1 (2.3)	0 (0.0)	42 (97.7)	43 (100.0)
48-59	0 (0.0)	0 (0.0)	55 (100.0)	55 (100.0)	0 (0.0)	0 (0.0)	36(100.0)	36 (100.0)
								
								
**Sex**		< 12.5cm	≥12.5cm			< 12.5cm	≥12.5cm	
Male		3 (1.8)	163 (98.2)	166(100.0)		10 (8.7)	105(91.3)	115(100.0)
Female		1 (0.8)	118 (99.2)	119(100.0)		6 (5.5)	103 94.5)	109(100.0)

MUAC = Mid- upper arm circumference, CIMCI = Community Integrated Management of Childhood Illness, LGA= Local Government Area

**Figure 1:**
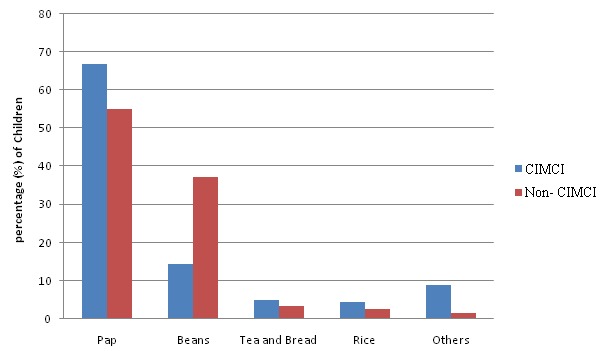
Types of Complementary Feeds

Most of the children commenced complementary feeding between 6-8 months of age in both LGAs. However more caregivers (19.3%) in the non-implementing area introduced complementary feeding earlier than 6 months and the difference was statistically significant (p < 0.001). On feeding of children less than two years about 89.7% of the children less than 6 months in the implementing LGA and 67.9% of those in non-implementing LGA feed more than 8 times in 24 hours. The difference was statistically significant (χ^2^=4.80, p= 0.028). Over 86% of children age 6-23 months in the implementing LGA feed more than three times a day, while more than half of children (52.1%) in the non-implementing LGA feed less than three times a day. The difference was statistically significant (χ^2^=31.3, p < 0.001) (Table 4). Concerning complementary feeding of children 6-9 months, pap was the most common food used for complementary feeding in both LGA, about 69% in the implementing area and 57% in the non-implementing area. In no instance was pap given alone by the caregivers in both LGA. The most common additives were powdered milk and sugar. Beans, rice, tea and bread are also used for complementary feeding. Others (fish and meat) are less common (Figure 1).

**Table 4:** Feeding practices of children less than two years by LGA

Variable	CIMCI implementing LGA n (%)	NON-CIMCI LGA n (%)	Statistical Indices
**Age (in Month) at introducing complementary food**	**N= 361**	**N = 361**	
<5	14 (4.2)	59 (19.3)	**χ^2^ =39.41, df = 2 P < 0.001
6-8	315 (94.9)	239 (78.1)	
>8	3 (0.9)	11(1.7)	
Total	332 (100.0)	306 (100.0)	
**Frequency of breastfeeding in 24 hours among < 6months** *	**N = 29**	**N = 53**	
**1-7** times	3 (10.3)	17 (32.1)	χ^2^=4.80, df = 2 P= 0.028
> 8 times	26 (89.7)	36 (67.9)	
Total	29 (100.0)	53 (100.0)	
**Breastfed children 6-23 months feeding 3 or more times a day** *	**N = 108**	**N = 106**	
Yes	93 (86.1)	70 (47.9)	χ^2^=31.33,df = 1 P< 0.001
No	15 (13.9)	76 (52.1)	
Total	108 (100.0)	106 (100.0)	

* Only children in the selected age subgroup were included in analysis.

** Likelihood Chi square test CIMCI = Community Integrated Management of Childhood Illness, LGA= Local Government Area

## Discussion

Nutritional status is an integral part of overall health of children and an indicator of well being of a child. In this study more children in the non implementing LGA (16.1%) had low weight for age compared to those in the CIMCI implementing LGA (3.6%). This difference was statistically significant (P = 0.000). Further more, the proportion of children with Low weight for age had also reduced from a baseline value of 20% pre CIMCI [13] to 3.6% in the CIMCI-implementing LGA in this study. This finding agrees with that from a study in Honduras assessing the impact of CIMCI on nutritional status of children. In their study the prevalence of severe malnutrition in the intervention area dropped remarkably from the initial level within two years of implementation of CIMCI similar to findings in this study [8, 14]. In all age groups a higher proportion of children had “low weight for age” in the non-implementing LGA than in the implementing LGA. In this study 17.1% of children less than one year in the implementing LGA had low weight for age compared to 30.7% in the non-implementing LGA. It can therefore be inferred that the difference in nutritional status of children observed between the two LGAs might not be unconnected with CIMCI implementation. A high proportion of low weight for age was observed in children 12- 23 months of age in the non-implementing LGA and this may not be unrelated with weaning and feeding practices of caregivers. Children under 23 months in the implementing LGA had better nutritional status than their peers in the non-implementing LGA. Children in the CIMCI implementing LGA had better MUAC measurement (>12.5cm) than their peers in non-implementing LGA for all age group. The MUAC was strongly correlated with the weight for age measurement of index children (r =0.70, p < 0.001). Children with low weight for age also had MUAC less than 12.5cm and were either moderately or severely malnourished. For all age groups and in all parameters children in the implementing LGA had better nutritional status than non-implementing LGA, thus further agreeing with the Honduras study which established that if improvements were sustained children in the intervention communities continued to do better than peers in comparison communities [8].

Appropriate timing of complementary feeding is vital in sustenance and improvement of nutritional status of children. In this study appropriate timing of complementary feeding was poor in the non-implementing LGA. The study found that a significantly higher (p < 0.001) percentage of caregiver in the non-implementing LGA (19.3%), commenced complementary feeding earlier than the recommended 6 months compared to just 4.2% in the implementing LGA. This is similar to findings by Vaahtera et al in rural Malawi [15] who observed that feeds were introduced much earlier than the recommended age. This finding also agrees with finding by Ebeuhi [13] in a previous study in Nigeria. Introduction of complementary feeding earlier than recommended has far-reaching implications for child survival, growth and development, as it denies the child the full benefits of EBF, and predisposes the child to increased risk of infection and malnutrition, amongst others [13]. In this study we found that nine in ten of caregivers in the implementing LGA introduced complementary feeding between the ages of 6-8 months in keeping with findings by UNICEF [16]. Pap was the commonest complementary feed in both LGA but often unfortified. Plain pap provides poor quality food for a child at a critical period of rapid growth and brain development. Fortified pap will be of benefit rather than the plain pap commonly used in feeding children and may possibly explain why there were more underweight children 30.7% amongst children less than 12 months in the non-implementing LGA. There is a need to improve upon the practice of giving poor quality food to children by educating the communities on the use of appropriate quantity and quality of complementary foods and also on right timing for introduction of such meal.

## Conclusion

The study demonstrated that children in the CIMCI implementing LGA had better nutritional status than there peer in the non-CIMCI implementing LGA, thus signifying the effectiveness of community level nutritional counseling as obtained in CIMCI. Proper communication and demonstration aimed at improving feeding practices are vital in improving nutritional status of children.
